# Physio-Transcriptomic Mechanism of Antimony Tin Oxide Nanoparticle-Induced Midgut Toxicity in *Bombyx mori*

**DOI:** 10.3390/biology15060508

**Published:** 2026-03-22

**Authors:** Yang Fang, Xuan Li, Fengchao Zhang, Yang Liu, Liang Ma, Liping Chen, Qijun Xie

**Affiliations:** 1 Hunan Key Laboratory of Biomedical Nanomaterials and Devices, School of Biological Science and Medical Engineering, Hunan University of Technology, Zhuzhou 412007, China; fangyang@hut.edu.cn (Y.F.); lixuan@stu.hut.edu.cn (X.L.); claralin527@hut.edu.cn (L.M.); 2507100202010@stu.hut.edu.cn (L.C.); 2 College of Marine Life Sciences, Ocean University of China, Qingdao 266003, China; 11210611009@stu.ouc.edu.cn (F.Z.); liuyang9573@stu.ouc.edu.cn (Y.L.)

**Keywords:** *Bombyx mori*, antimony tin oxide, oxidative stress, midgut, RNA sequencing, physiological impairment

## Abstract

The silkworm (*Bombyx mori*) is an economically important insect that plays a crucial role in agricultural development. Antimony tin oxide, a high-tech multifunctional nanomaterial, is extensively utilized in contemporary industries due to its properties of transparency, conductivity, and stability. Nevertheless, the toxicity and potential adverse effects of antimony tin oxide on living organisms remain poorly understood. In this study, we evaluated the effects of antimony tin oxide at varying concentrations (0–3.2 μg/μL) on the growth, oxidative stress response, gene expression, and midgut integrity of fifth-instar silkworm larvae. Exposure to high concentrations of antimony tin oxide resulted in a significant reduction in larval weight and severely disrupted the antioxidant defense system. RNA sequencing (RNA-Seq) analysis identified 239 differentially expressed genes (DEGs), which were confirmed by qPCR, revealing up-regulated lipid synthesis gene *AGPAT5*, down-regulated chitin degradation gene *Chi*, and suppressed glycerolipid hydrolysis gene *H9J6N7_BOMMO*. Histopathological and ultrastructural examinations revealed severe damage to the structure of midgut epithelial cells. Structural and functional analysis of conserved domains in key DEG-encoded proteins revealed that gene dysregulation disrupted energy metabolism and compromised the physical barrier, ultimately linking molecular abnormalities to observed tissue damage. These findings elucidate the mechanisms by which antimony tin oxide induces midgut toxicity through interference with critical metabolic pathways and functional perturbations at the molecular level.

## Simple Summary

Antimony tin oxide nanomaterials are widely used in industry, but their biological toxicity remains unclear. This study investigated the effect of antimony tin oxide on silkworms (*Bombyx mori*), an economically important insect, with a focus on larval growth and midgut health. Exposure to high antimony tin oxide concentrations (up to 3.2 μg/μL) significantly reduced larval weight, damaged midgut cell structures, and impaired antioxidant defenses. RNA-seq analysis identified 239 dysregulated genes validated by qPCR, upregulating lipid synthesis (*AGPAT5*), down-regulating chitin degradation (*Chi*), and suppressing glycerolipid hydrolysis (*H9J6N7_BOMMO*). These findings reveal the toxic mechanisms of antimony tin oxide through metabolic interference, highlight potential risks to sericulture, and urge environmental caution in nanomaterial applications.

## 1. Introduction

The rapid advancement of nanotechnology has facilitated the extensive application of engineered nanomaterials (ENMs) across a diverse range of industrial and biomedical sectors. Due to their distinctive physicochemical properties, ENMs substantially enhance the performance of foundational materials and introduce innovative functionalities. These materials are widely employed in both veterinary and human medicine [1,2,3,4,5], aquaculture [6,7,8], livestock production [9], environmental remediation [10,11], water treatment [12,13], and the food industry [14,15,16]. Nonetheless, the pervasive utilization of ENMs has led to their unavoidable release into the environment, thereby posing potential ecological risks. The precise monitoring of environmental concentrations and the assessment of the toxicity of these materials remain formidable challenges.

Antimony tin oxide is a functional nanomaterial recognized for its transparency and electrical conductivity. In addition to its well-documented applications in biological and electrochemical devices [17,18], solar cells [19], and smart windows [20], antimony tin oxide exhibits emerging potential for enhancing wastewater treatment and air purification systems [21]. Furthermore, antimony tin oxide nanoparticles have been investigated as substrates for surface-enhanced Raman scattering (SERS) [22]. Despite their industrial significance, research on the toxicity and environmental impact of antimony tin oxide nanoparticles remains limited [23].

The silkworm, a well-established model organism within the Lepidoptera order, presents unique advantages for toxicological evaluations, drug discovery, and developmental biology research. Its utility is attributed to its rapid life cycle, straightforward husbandry requirements, and a fully annotated genome. The significance of *Bombyx mori* extends beyond its traditional role in sericulture, encompassing applications in environmental risk assessment and nanotoxicology [24,25]. The midgut of *Bombyx mori*, as the principal site for nutrient digestion and absorption, also serves as a primary barrier against xenobiotics [26]. Structural and functional impairments of the midgut have been shown to negatively impact silkworm growth and development, as evidenced by studies involving insecticides such as indoxacarb [27], cyantraniliprole [28], and microplastics [29,30]. However, comprehensive investigations into the toxicological effects and molecular mechanisms of antimony tin oxide on silkworms remain limited.

To address this knowledge gap regarding biological toxicity of antimony tin oxide, the present study was designed to investigate its effects on fifth-instar silkworm larvae. Specifically, we aimed to characterize the physiological responses (growth and oxidative stress), transcriptional alterations, histopathological changes in the midgut, and structural and functional conservation analyses of proteins following antimony tin oxide exposure. Our results demonstrate that the toxicity of antimony tin oxide is mediated through the dysregulation of metabolic pathways and structural damage to the midgut epithelium. This work elucidates the underlying mechanisms of antimony tin oxide-induced toxicity, thereby filling a critical gap in the assessment of its environmental safety.

## 2. Materials and Methods

### 2.1. Silkworm Strain and Rearing

The silkworm larvae, specifically the Chufeng × Hanyun strain, were sourced from the Economic Crops Research Institute of the Hubei Academy of Agricultural Sciences. The rearing environment was meticulously controlled, maintaining a temperature of 25 ± 1 °C and a relative humidity ranging from 70% to 75%. Each larva in the fifth instar was individually housed within a Petri dish, with all dishes collectively situated in a constant-temperature incubator. The larvae were nourished with fresh mulberry leaves and were subjected to a 12 h light–dark cycle.

### 2.2. Experimental Procedure

Commercial antimony tin oxide nanoparticles (Macklin, A801372, Shanghai, China) were used in this study. According to the supplier’s specifications, the primary particle size is approximately 20–80 nm and the purity is >99.5%. The material was dispersed in ultrapure water and homogenized using an ultrasonic processor prior to exposure preparation. Independent characterization of hydrodynamic particle size, zeta potential, morphology, and colloidal stability under the present exposure conditions was not performed in this study. The control group received mulberry leaves treated solely with ultrapure water. Subsequently, various concentrations of antimony tin oxide (0.1, 0.4, 0.8, 1.6, and 3.2 μg/μL) were prepared. Each concentration was allocated to a separate, clean glass container. Fresh, healthy mulberry leaves were fully immersed in the antimony tin oxide suspensions to ensure uniform coating and were then air-dried at room temperature for 2 h prior to being administered to the larvae. A feeding experiment was conducted using fifth-instar larvae, with three replicates established for each concentration; each replicate comprised ten silkworms. On the initial day of the experiment, each group was fed mulberry leaves treated with a designated antimony tin oxide concentration, with each larva receiving 5 g of treated leaves to ensure adequate intake. To ensure quantitative daily intake of treated leaves, each larva was individually fed. The 5 g portion per larva was completely consumed during each feeding session, guaranteed by preliminary consumption tests. This approach is consistent with established oral exposure methods used in insect toxicology, where precise control of ingested dose is achieved by providing defined quantities of treated diet that are fully consumed [31]. Prior to each subsequent feeding, any remaining leaves and deceased larvae were removed. Initial larval weights and mortality rates were recorded before the first feeding, and these parameters were monitored regularly until the larvae reached maturity and initiated cocoon formation.

### 2.3. Body Weight of Silkworms

The body weights of the silkworms were systematically measured and documented daily at predetermined intervals. Upon reaching maturation and initiating cocoon formation, the larvae were manually collected and transferred into disposable containers to complete the spinning process. Following the completion of spinning, three mass parameters were quantified: composite cocoon weight, endopupal weight, and structural shell weight. Subsequently, the cocoon shell rate was calculated for each group using the formula: (cocoon shell weight/cocoon weight) × 100%.

### 2.4. Measurement of Reactive Oxygen Species (ROS) Levels, Superoxide Dismutase (SOD) Activity, Catalase (CAT) Activity, and Glutathione (GSH) Content

Based on the initial concentration-screening experiment, 3.2 μg/μL was selected as the representative high-dose exposure for oxidative stress assays because the Kaplan–Meier survival analysis showed that this concentration caused approximately 50% larval mortality during the observation period under our experimental conditions, while still allowing sufficient surviving individuals for downstream mechanistic analyses.

The level of reactive oxygen species (ROS) was measured based on the reaction of hydroxyl radicals with the oxidation-sensitive probe 2,7-dichlorofluorescein diacetate (DCFH-DA) (Applygen, Beijing, China). Upon reaction with ROS, DCFH-DA is oxidized to the fluorescent compound DCFH, which was quantified using an FLX 800 fluorescence microplate reader (Bio-Tek, Winooski, VT, USA). Body tissues from the high-concentration treatment group (3.2 μg/μL) and the control group were flash-frozen in liquid nitrogen, homogenized, and mixed with an equal volume of phosphate-buffered saline (PBS). Each group consisted of four biological replicates. Homogenates underwent 3 min vortex mixing followed by centrifugation (2800× *g*, 3 min). Then, 30 μL of the supernatant was collected and diluted to a final volume of 100 μL with PBS. Subsequently, 100 μL of the diluted sample was mixed with 100 μL of DCFH-DA (30 μmol/L) and transferred into microplate wells. The plate was incubated at 37 °C in complete darkness for 30 min. Fluorescence was then measured using a microplate reader (Synergy H1, BioTek, Winooski, VT, USA) at excitation and emission wavelengths of 485 nm and 520 nm, respectively. ROS levels were quantified and expressed as units per milligram of protein (U/mg protein).

To determine superoxide dismutase (SOD) activity, 18 µL of the supernatant was combined in a 96-well microplate with 48 µL of PBS, 12 µL of xanthine oxidase, 36 µL of a solution containing 0.037 U/L nitroblue tetrazolium (NBT), 6 µmol/L xanthine, and 68 µmol/L salicylic acid. Following incubation at 37 °C for 30 min, SOD activity was measured at an absorbance of 560 nm using a microplate reader. Enzyme activity was expressed in units per milligram of protein (U/mg protein).

For the catalase (CAT) activity assay, 20 µL of the supernatant was mixed with 100 µL of a reaction solution containing 20 mmol/L hydrogen peroxide. The mixture was incubated at room temperature for 10 min. Subsequently, 180 µL of a stop reagent containing 50 mmol/L ammonium molybdate tetrahydrate was added. CAT activity was then measured spectrophotometrically at 405 nm using a microplate reader and expressed as units per milligram of protein (U/mg protein).

To assess the glutathione (GSH) content, 20 µL of the supernatant was combined with 100 µL of a 0.1 mmol/mL DTNB solution and incubated at room temperature in the absence of light for 5 min. Subsequently, 180 µL of a 0.1 mol/L phosphate buffer (pH 7.4) was added. The GSH content was then quantified by measuring the absorbance at 412 nm.

The activities of superoxide dismutase (SOD) and catalase (CAT), as well as the content of glutathione (GSH), were determined using commercial assay kits from the Nanjing Jiancheng Bioengineering Institute (Nanjing, China), following the manufacturer’s instructions.

### 2.5. RNA-Seq and Bioinformatics Analysis

Total RNA was extracted from midgut tissues using RNAiso Plus (Takara, Dalian, China) and subsequently quantified with a NanoDrop-2000 spectrophotometer (Thermo Fisher Scientific, Waltham, MA, USA). The integrity of the RNA was assessed via agarose gel electrophoresis. High-quality RNA samples were selected for complementary DNA (cDNA) library construction utilizing the MGIEasy RNA Sample Prep kit (BGI, Shenzhen, China), in accordance with the manufacturer’s instructions. Sequencing was conducted on the DNBSEQ™ platform (BGI, Shenzhen, China), yielding 150 base pair paired-end reads. HISAT2 (version 2.2.1) was used to align the reads to the *Bombyx mori* reference genome (BmDZ.v3.6.gff3). Transcript expression levels were quantified in fragments per kilobase of transcript per million mapped reads (FPKM). Differentially expressed genes (DEGs) were defined as those with FDR < 0.05 and |log2 fold change| > 1. To elucidate the biological significance, the identified DEGs were subjected to enrichment analyses for Gene Ontology (GO).

### 2.6. Reverse Transcription Quantitative Real-Time PCR (RT-qPCR)

Fifth-instar larvae from both the control group and the 3.2 µg/µL antimony tin oxide-treated group were sampled. Total RNA was isolated from whole-body tissues using TRIzol reagent (Thermo Fisher Scientific) following the manufacturer’s protocol. First-strand cDNA synthesis was performed with approximately 1–2 µg of total RNA using the HiScript IV All-in-One Ultra RT SuperMix for qPCR (Vazyme, Nanjing, China) with oligo (dT) primers at 42 °C for 60 min. Gene-specific primers were designed based on transcriptomic data (Table S2). qPCR was performed using a LightCycler 96 system (Roche, Basel, Switzerland) or a QuantGene9600 system using the 2 × SYBR Green qPCR Master Mix (Vazyme, Nanjing, China) to quantify expression levels relative to the rp49 reference gene. The amplification protocol consisted of an initial denaturation at 95 °C for 3 min, followed by 40 cycles of 95 °C for 10 s, 58 °C for 20 s, and 72 °C for 20 s, with subsequent melting curve analysis over 55 °C to 98 °C. Relative transcription changes were calculated via the 2^−ΔΔCT^ method, incorporating three biological replicates and two technical replicates per sample.

### 2.7. Histopathological Examination

Midgut tissues from each group were fixed in 4% paraformaldehyde and embedded in paraffin blocks. Sections were cut at a thickness of 5 µm and mounted onto glass slides. After staining with hematoxylin and eosin (HE), the tissue sections were examined and imaged using a Nikon DS-U3 imaging system (Nikon, Tokyo, Japan).

### 2.8. Transmission Electron Microscope (TEM)

On the fifth day, four fifth-instar larvae were collected from both the antimony tin oxide (3.2 μg/μL) treatment group and the control group. The midgut tissues were dissected from anesthetized larvae and immediately fixed overnight in 2.5% glutaraldehyde at 4 °C. The samples were then rinsed with 0.2 mol/L phosphate buffer (pH 7.4), post-fixed in 1% osmium tetroxide for 1 h, and dehydrated through a graded ethanol series (75%, 85%, 95%, and 100%). Subsequently, the tissues were stained overnight with 0.5% uranyl acetate, embedded in Epon 812 resin, and further stained with lead citrate and uranyl acetate. Ultrathin sections (100 nm) were prepared using a Leica UC6 ultramicrotome and examined under an HT7700 transmission electron microscope (Hitachi, Tokyo, Japan).

### 2.9. Integrated Analysis of Protein Structural Features and Functional Networks

Protein sequences of key enzymes from three energy metabolism pathways (fatty acid biosynthesis, amino sugar/nucleotide sugar metabolism, and glycerolipid synthesis) were retrieved. Their conserved motifs were identified using the MEME Suite (version 5.5.7) with default parameters. The distribution of identified motifs was visualized using TBtools (v2.119). Sequence logos representing conserved residues were generated using WebLogo 3. Additionally, functional domains were predicted using the CD-Search tool (CDD v3.20, https://www.ncbi.nlm.nih.gov/Structure/cdd/wrpsb.cgi, accessed on 20 December 2025) available at NCBI.

### 2.10. Computational Proteomics: Structure, PPI and GO Analysis

To investigate structural conservation, homology-based tertiary structure modeling of AGPAT5, Chi, and H9J6N7_BOMMO was performed using SWISS-MODEL (https://swissmodel.expasy.org/, accessed on 19 January 2026). Protein structural similarity was quantitatively assessed using three metrics: sequence similarity, TM-score, and RMSD. Sequence similarity was calculated through pairwise sequence alignment using the Needleman–Wunsch algorithm available on NovoPro (https://www.novopro.cn/tools/needle.html, accessed on 19 January 2026). Tertiary structure alignments were conducted with TM-align (https://zhanggroup.org/TM-align/, accessed on 19 January 2026) to derive TM-score and RMSD values. To explore functional interactions, a PPI network was constructed, followed by Gene Ontology (GO) enrichment analysis using the STRING database (https://cn.string-db.org/, accessed on 19 January 2026).

### 2.11. Statistical Analysis

Data are presented as mean ± SD. Comparisons between two groups were performed using Student’s *t*-test. For experiments involving more than two groups, one-way ANOVA followed by Dunnett’s multiple-comparison test was used to compare each treatment group with the control. The survival differences between the treatment groups were statistically analyzed using the log-rank test. Differences were considered statistically significant at *p* < 0.05.

## 3. Results

### 3.1. Effect of Antimony Tin Oxide Concentration on Silkworm Body Weight Gain

**Figure 1:**
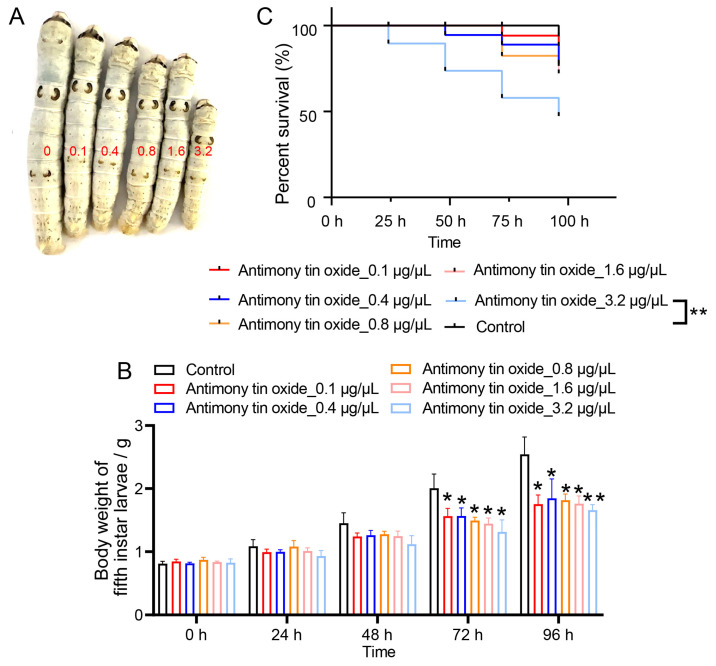
The effect of different concentrations of antimony tin oxide on the weight gain of silkworms: (**A**) Morphological changes in fifth-instar silkworm larvae after 96 h of treatment with different concentrations of antimony tin oxide. (**B**) The effect of different concentrations of antimony tin oxide treatment on the weight changes in silkworm fifth-instar larvae over time. (**C**) Kaplan–Meier survival curves of larvae exposed to different concentrations of antimony tin oxide during the observation period (*n* = 30 per group). Survival differences were analyzed using the log-rank test. * *p* < 0.05, ** *p* < 0.01.

To evaluate the effect of different concentrations of antimony tin oxide on silkworm weight gain, fifth-instar larvae were fed mulberry leaves treated with varying concentrations of antimony tin oxide, and their weights were recorded at 0, 24, 48, 72, and 96 h. Silkworms fed antimony tin oxide-treated leaves exhibited smaller body sizes compared to those of the control group, with a more pronounced reduction observed at higher antimony tin oxide concentrations (Figure 1A). Our data further demonstrate that weight gain was inhibited in silkworms exposed to antimony tin oxide, and this inhibitory effect intensified with both increasing antimony tin oxide concentration and exposure time (Figure 1B). In conclusion, antimony tin oxide suppressed weight gain in fifth-instar silkworm larvae in a concentration-dependent manner. Given that 3.2 μg/μL caused the most pronounced toxic phenotype and resulted in approximately 50% larval mortality according to the Kaplan–Meier survival curve, while remaining non-completely lethal, this concentration was selected for subsequent mechanistic analyses (Figure 1C).

### 3.2. Antimony Tin Oxide-Induced Oxidative Stress in Silkworms

**Figure 2:**
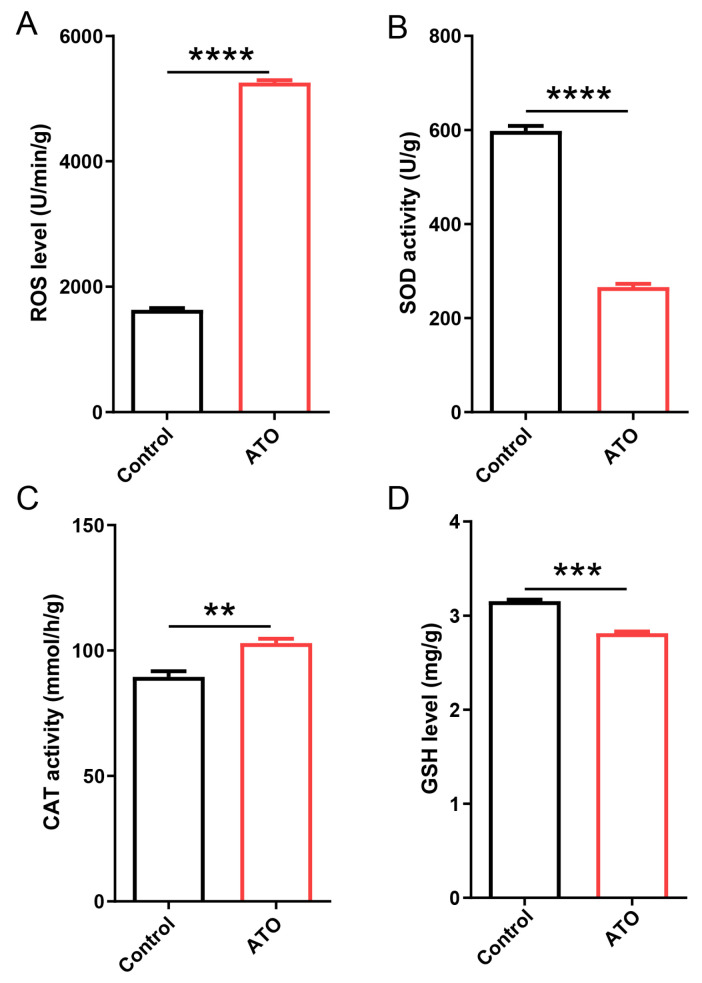
Oxidative stress parameters in silkworms after antimony tin oxide exposure. (**A**) 3.2 μg/μL of antimony tin oxide significantly increased ROS levels. (**B**) 3.2 μg/μL of antimony tin oxide significantly reduced SOD activity. (**C**) 3.2 μg/μL of antimony tin oxide enhanced CAT activity. (**D**) 3.2 μg/μL of antimony tin oxide significantly reduced GSH content. ** *p* < 0.01, *** *p* < 0.001, **** *p* < 0.0001.

To elucidate the toxic mechanism of antimony tin oxide, key oxidative stress-related indicators were measured. In fifth-instar larvae treated with 3.2 μg/μL antimony tin oxide, the level of ROS increased significantly compared to the control group, indicating severe oxidative stress (Figure 2A). Concurrently, SOD activity markedly decreased under the same treatment conditions (Figure 2B). While high-concentration antimony tin oxide exposure increased CAT activity, it also induced substantial depletion of GSH (Figure 2C,D). These findings collectively demonstrated that antimony tin oxide disrupted the antioxidant defense system, leading to oxidative damage in silkworm larvae.

### 3.3. RNA-seq Analysis of the Effects of Antimony Tin Oxide on Silkworm Gene Expression

**Figure 3:**
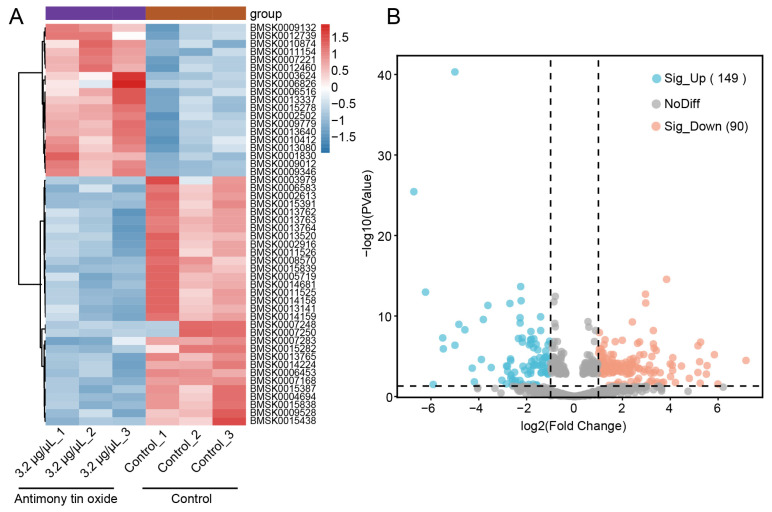
mRNA expression changes revealed by RNA-seq in silkworms exposed to antimony tin oxide (3.2 μg/μL). (**A**) Heatmap of DEGs between the control group and the 3.2 μg/μL antimony tin oxide treatment group, with colors representing relative expression levels. (**B**) The volcano plot was used to analyze the transcriptional differences between the 3.2 μg/μL antimony tin oxide treatment group and the control group.

Transcriptomic analysis of midgut tissues from larvae exposed to 3.2 μg/μL antimony tin oxide revealed widespread changes in gene expression (Figure 3A). Differential expression analysis identified 239 DEGs, comprising 149 upregulated and 90 down-regulated genes (Figure 3B, Supplementary Table S1). GO and KEGG enrichment analyses were subsequently performed to elucidate the potential functions of these DEGs.

### 3.4. Antimony Tin Oxide Reprograms Silkworm Oxidation-Metabolism Pathways

**Figure 4:**
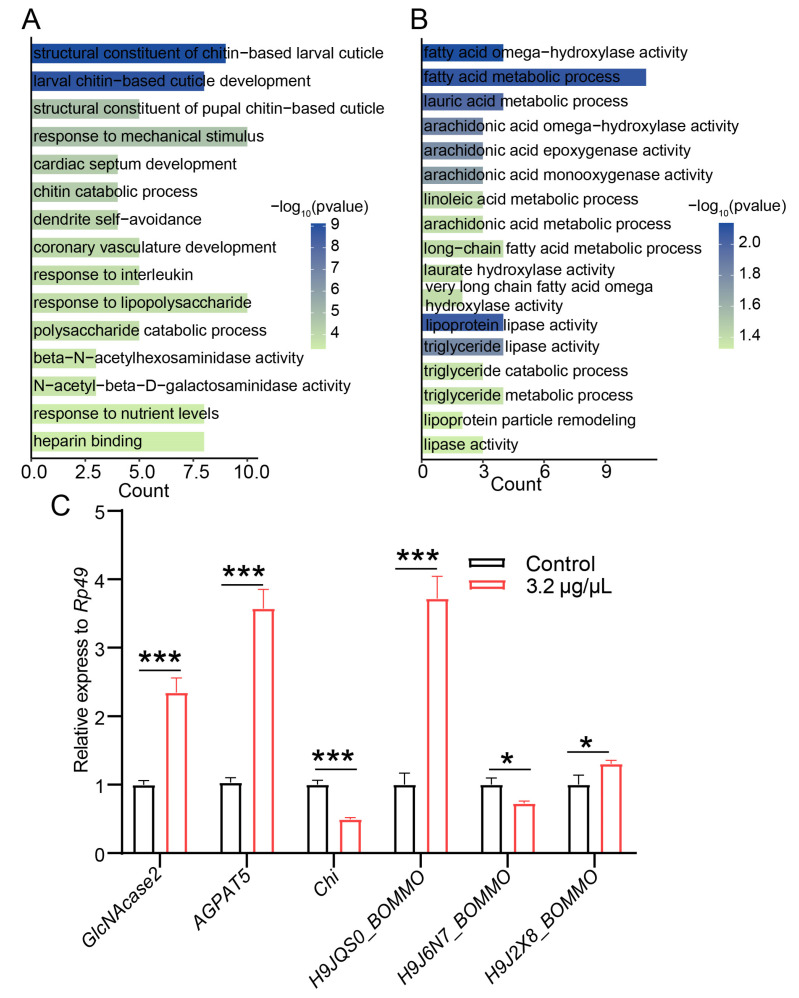
GO enrichment analysis and RT-qPCR validation of differentially expressed genes in the midgut of *Bombyx mori* larvae following antimony tin oxide exposure. (**A**) GO enrichment analysis of upregulated genes. (**B**) GO enrichment analysis of down-regulated genes. (**C**) qRT-PCR validation of selected antimony tin oxide-responsive DEGs. Glycolysis/Gluconeogenesis (*GlcNAcase2*), fatty acid metabolism (*AGPAT5*), amino sugar and nucleotide sugar metabolism (*Chi*), glycerolipid metabolism (*H9JQS0_BOMMO*, *H9J6N7_BOMMO*), cholesterol metabolism and glycine/serine/threonine metabolism (*H9J2X8_BOMMO*). * *p* < 0.05, *** *p* < 0.001.

GO enrichment analysis of the 239 DEGs revealed distinct functional patterns between the up- and down-regulated gene sets (Figure 4A,B). The up-regulated DEGs were mainly enriched in stress defense and structural maintenance-related categories, including structural constituent of cuticle, chitin metabolic process, response to mechanical stimulus, and immune response-related terms. In contrast, the down-regulated DEGs were primarily enriched in lipid metabolic processes, such as fatty acid metabolic process, regulation of ω-oxidation, triacylglycerol metabolism, and metabolic enzyme activities.

To validate the RNA-seq data, six representative DEGs were examined by RT-qPCR (Figure 4C). The results showed that *GlcNAcase2* and *AGPAT5* were significantly up-regulated, whereas *Chi* and *H9J6N7_BOMMO* were markedly down-regulated. In addition, *H9J2X8_BOMMO* and *H9JQS0_BOMMO* also showed increased expression. Overall, the RT-qPCR results were consistent with the transcriptomic trends, supporting the reliability of the RNA-seq dataset.

Together, these results indicated that antimony tin oxide exposure was associated with significant alterations in genes involved in cuticle/chitin-related processes and lipid metabolism, suggesting a substantial remodeling of midgut metabolic and structural response pathways.

### 3.5. Histological Observation of the Silkworm Midgut by HE Staining

**Figure 5:**
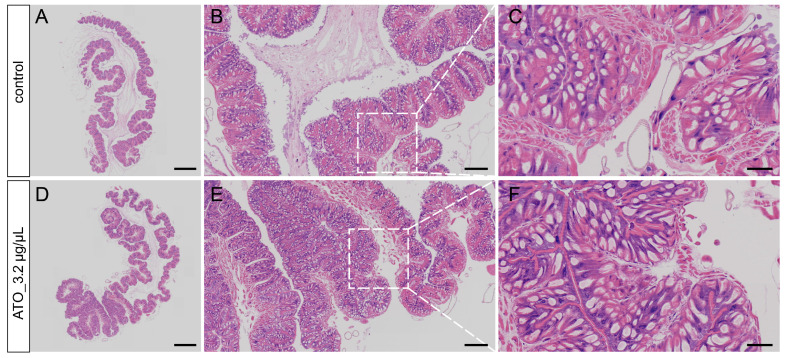
Histopathological examination of the midgut of silkworm larvae exposed to antimony tin oxide. (**A**–**C**) The pathological evaluation of the midgut in the control group. (**D**–**F**) The pathological evaluation of the midgut in the antimony tin oxide treatment group. (**C**,**F**) is the enlarged view of the white boxed area in (**B**,**E**). Scale bar: 1000 μm (**A**,**D**), 200 μm (**B**,**E**), 50 μm (**B**,**E**).

To assess the effect of antimony tin oxide on midgut structure in silkworms, we used HE staining. As shown in Figure 5A–C, the intestinal wall structure of the control group was complete, with closely packed and tightly arranged cells and normal cell morphology. In contrast, the intestinal wall cell layer of the antimony tin oxide treatment group was thinner, the cells were loosely arranged, and the cell gaps were enlarged (Figure 5D–F). At higher magnification, the muscle layer of the intestinal wall was damaged in the antimony tin oxide-treated group (Figure 5F) compared with the control group. These results confirmed that antimony tin oxide would cause structural and functional abnormalities in the midgut.

### 3.6. Midgut Organelle Remodeling in Antimony Tin Oxide-Exposed Silkworms

**Figure 6:**
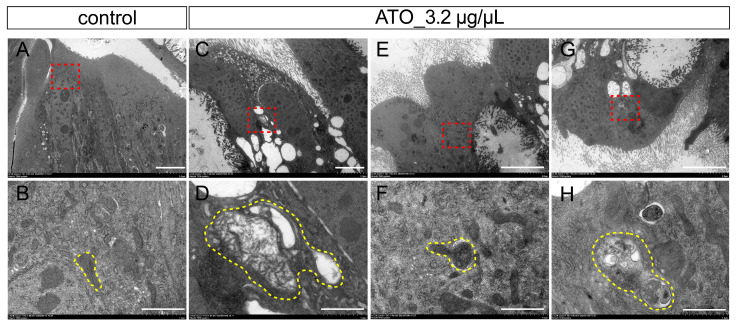
Ultrastructural analysis of the midgut cells in silkworm larvae exposed to antimony tin oxide. (**A**,**B**) The ultrastructural observation of the midgut cells in the control group, and (**B**) is the enlarged view of the red boxed area in (**A**). (**C**,**D**) The ultrastructural observation of the midgut cells in the antimony tin oxide treatment group, and (**D**) is the enlarged view of the red boxed area in (**C**). (**E**,**F**) The ultrastructural observation of autophagic vesicles in the antimony tin oxide treatment group, and (**F**) is the enlarged view of the red boxed area in (**E**). (**G**,**H**) The ultrastructural observation of autophagosomes in the antimony tin oxide treatment group, and (**H**) is the enlarged view of the red boxed area in (**G**). Yellow dashed lines indicate mitochondria and autophagic vesicles. Scale bar: 5 μm (**A**,**C**), 1 μm (**B**,**D**–**H**).

The ultrastructure of midgut organelles in silkworms was observed by TEM. In the control group, the mitochondria of midgut cells were intact and microvilli were densely and evenly distributed (Figure 6A,B). In the antimony tin oxide treatment group, the structure of midgut cell mitochondria changed significantly, showing mitochondrial swelling, cristae rupture, and a large number of vacuoles (Figure 6C,D). Autophagosomes were also observed (Figure 6G,H), and the phenomenon of autophagosomes engulfing mitochondria was also observed (Figure 6E,F). These results further confirmed that antimony tin oxide could cause structural and functional abnormalities of midgut organelles. These results further confirmed that antimony tin oxide could cause abnormalities in the structure and function of the midgut organelles.

### 3.7. Functional Motif Signatures in Metabolic Gene Evolution

**Figure 7:**
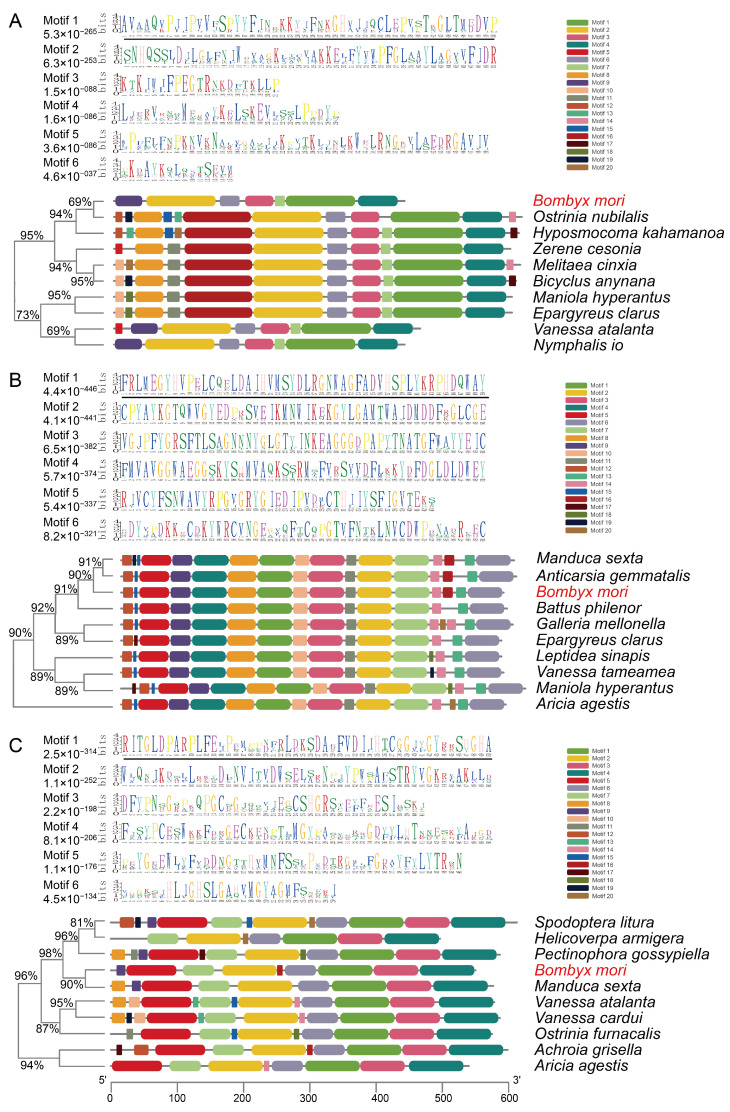
Motif analysis of three metabolic pathway proteins and their orthologous sequences. (**A**) AGPAT5 and orthologs, (**B**) Chi and orthologs, (**C**) H9J6N7_BOMMO and orthologs. Conserved motifs are represented by numbered colored boxes. Black horizontal lines denote consensus amino acid sequences. The insect species abbreviations are as follows: AGPAT5, Chi, and H9J6N7_BOMMO: *Bombyx mori*, XP_034824087.1: *Maniola hyperantus*, XP_038209095.1: *Zerene cesonia*, XP_072936226.1: *Epargyreus clarus*, XP_026323669.1: *Hyposmocoma kahamanoa*, XP_047539514.1: *Vanessa atalanta*, XP_050359267.1: *Nymphalis io*, XP_045452641.1: *Melitaea cinxia*, XP_063837193.1: *Ostrinia nubilalis*, XP_023939060.1: *Bicyclus anynana*, XP_030030175.1: *Manduca sexta*, XP_050672426.1: *Leptidea sinapis*, XP_052754063.1: *Galleria mellonella*, XP_026497290.2: *Vanessa tameamea*, XP_075984553.1: *Anticarsia gemmatalis*, XP_041982984.1: *Aricia agestis*, XP_072933371.1: *Epargyreus clarus*, XP_034836040.1: *Maniola hyperantus*, XP_068619839.1: *Battus philenor*, XP_030027522.1: *Manduca sexta*, XP_022814449.1: *Spodoptera litura*, XP_049868595.1: *Pectinophora gossypiella*, XP_049696367.2: *Helicoverpa armigera*, XP_059046451.1: *Achroia grisella*, XP_028173132.1: *Ostrinia furnacalis*, XP_047530437.1: *Vanessa atalanta*, XP_046962056.1: *Vanessa cardui*, XP_041982175.1: *Aricia agestis*.

Bioinformatic analysis revealed that *Bombyx mori* proteins AGPAT5, Chi, and H9J6N7_BOMMO share sequence similarities of 72.50%, 84.36%, and 62.27% with their orthologs in other species, respectively (Figure 7, Supplementary Figures S1–S3 and Supplementary Table S3). MEME Suite analysis identified a conserved PIsC domain in AGPAT5, a key enzyme in fatty acid metabolism, which catalyzes phosphatidic acid synthesis as the core precursor for glycerophospholipid biosynthesis on the endoplasmic reticulum (Figure 7A) [32]. Its upregulation aligns with enhanced membrane lipid production required for epithelial regeneration and immune cell activation during intestinal repair [33].

Chi, a key enzyme in amino sugar and nucleotide sugar metabolism, possesses a conserved GH18 domain with dual chitinase functions: hydrolyzing chitin to generate bioactive oligosaccharides and acting as a pattern recognition receptor for non-hydrolytic chitin binding (Figure 7B) [34]. Its down-regulation suggests impaired peritrophic membrane maintenance and disrupted chitin metabolic homeostasis, exacerbating intestinal barrier dysfunction under stress [35], consistent with observed morphological damage.

Moreover, H9J6N7_BOMMO, a key enzyme in glycerolipid metabolism pathway, contains a conserved domain belonging to the carboxylesterase family. This enzyme hydrolyzes triglycerides and phospholipids to release free fatty acids and glycerol (Figure 7C) [36], and it may regulate lipoprotein assembly in hemolymph [37]. Its down-regulation implies digestive enzyme suppression, as organisms prioritize energy allocation toward repair over digestion during gut injury [38].

### 3.8. Evolutionary Conservation of Structural Domains and Functional Features of AGPAT5, Chi, and H9J6N7_BOMMO

**Figure 8:**
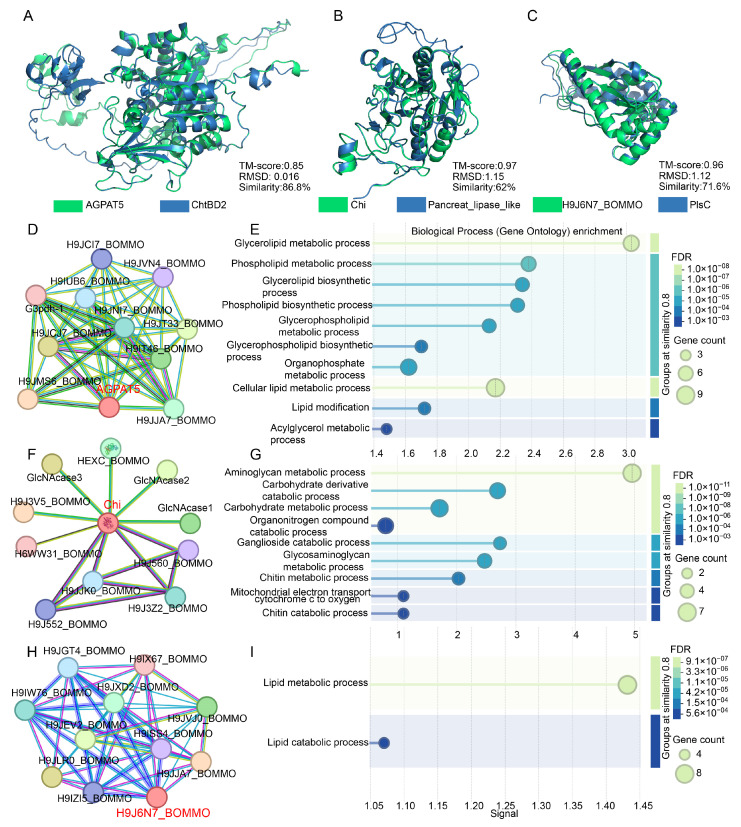
Structural and functional conservation analysis of AGPAT5, Chi, and H9J6N7_BOMMO proteins. (**A**–**C**) Comparative tertiary structures identify AGPAT5, Chi, and H9J6N7_BOMMO as structural orthologs of ChtBD2, Pancreat_lipase_like, and PIsC, respectively. Sequence similarity exceeding 50% indicates high conservation. TM-scores (range: 0–1; >0.5 indicates shared fold type) quantify structural resemblance. RMSD values < 1.5 Å confirm near-identical tertiary conformations. (**D**,**F**,**H**) Protein–protein interaction networks predicted via STRING database for AGPAT5, Chi, and H9J6N7_BOMMO. (**E**,**G**,**I**) Gene Ontology (GO) enrichment analysis (Significance threshold: FDR < 0.05).

To further evaluate their functions, we performed protein structure modeling and homology comparisons. Prediction results revealed that the tertiary structure of AGPAT5 shares 86.8% sequence similarity with the *Epargyreus clarus* homolog ChtBD2, with a template modeling score (TM-score) of 0.85 and a root mean square deviation (RMSD) of 0.016 Å (Figure 8A). Chi exhibited 62% sequence similarity to *Manduca sexta* homolog Pancreat_lipase_like, with a TM-score of 0.97 and an RMSD of 1.15 Å (Figure 8B). H9J6N7_BOMMO showed 71.6% sequence similarity to *Manduca sexta* homolog PIsC, with a TM-score of 0.96 and an RMSD of 1.12 Å (Figure 8C). These results suggest that AGPAT5, Chi, and H9J6N7_BOMMO likely possess functions similar to their homologs, indicating functional conservation.

Using STRING interaction data, we identified three functional modules in the midgut of silkworm under antimony tin oxide-induced oxidative stress. AGPAT5 formed a lipid-metabolism module, directly interacting with lipid-metabolizing enzymes (Figure 8D). As a 1-acyl-sn-glycerol-3-phosphate acyltransferase, it was involved in phospholipid synthesis and likely cooperates with glycerol-3-phosphate dehydrogenase (G3pdh-1) and related acyltransferases to regulate phosphatidic acid production under stress. Chi constituted a chitin-degradation core, interacting with multiple β-N-acetylhexosaminidases to form a complete pathway from chitin hydrolysis to N-acetylglucosamine generation (Figure 8F). This module linked chitin catabolism to downstream amino sugar metabolism. H9J6N7_BOMMO formed the center of a dietary lipid-processing module, interacting with lipases, acyltransferases, and phosphatases to balance triglyceride hydrolysis, diacylglycerol generation, and acyl remodeling (Figure 8H). Together, these modules highlighted key metabolic nodes in lipid synthesis, chitin degradation, and dietary lipid utilization during oxidative stress [39].

GO enrichment analysis further clarified their roles. The AGPAT5-associated network was enriched in glycerolipid metabolism (enrichment factor 3.0), supporting its function in phosphatidic acid synthesis and membrane lipid remodeling (Figure 8E). Chi showed specific enrichment in aminoglycan metabolism (enrichment factor 5.0), consistent with its GH18 domain mediating chitin hydrolysis and potential immune recognition (Figure 8G). H9J6N7_BOMMO was broadly associated with lipid metabolic processes (enrichment factor 1.5), reflecting its carboxylesterase domain’s role in integrating lipolysis and lipid remodeling (Figure 8I). Thus, AGPAT5 acted as a lipid-precursor supplier, Chi as a chitin-degradation and signaling hub, and H9J6N7_BOMMO as a multifunctional integrator of dietary lipid metabolism.

## 4. Discussion

Despite the widespread application of antimony tin oxide in high-technology industries, its ecological toxicity remains insufficiently understood, particularly in terrestrial and agriculturally relevant organisms. Although previous studies have examined the biological effects of antimony-containing materials in aquatic systems, evidence regarding their impact on economically important insects is still limited [40]. As a classical lepidopteran model with well-established use in toxicological and physiological research, *Bombyx mori* provides a useful system for evaluating the biological risks of engineered nanomaterials [24,25]. In the present study, we combined physiological, biochemical, histological, ultrastructural, and transcriptomic analyses to characterize the toxic effects of antimony tin oxide on fifth-instar silkworm larvae and to explore the potential mechanisms underlying midgut injury.

Our results demonstrated that dietary exposure to antimony tin oxide caused clear concentration-dependent toxicity in silkworm larvae. The most pronounced effects were observed in the 3.2 μg/μL treatment group, in which larval growth was markedly inhibited, body size was reduced, and survival declined substantially. These phenotypic changes indicate that antimony tin oxide exerts a strong adverse influence on larval development. Notably, exposure to 3.2 μg/μL antimony tin oxide resulted in severe growth inhibition and marked tissue damage. The inhibitory effect of antimony tin oxide on silkworm growth is consistent with previously documented effects of rare earth elements on insect development [41,42]. Similar growth retardation effects have been observed in other insect species exposed to rare earth elements, which are known to interfere with hormonal regulation and disrupt critical developmental processes such as molting and pupation [43]. These findings are in concordance with earlier reports on the high toxicity of indoxacarb to silkworms [27]. It should nevertheless be noted that the growth assay included only three biological replicates per concentration, which may have limited the statistical power to detect treatment effects at the 48 h time point.

Further investigation into oxidative stress responses revealed that antimony tin oxide induced severe oxidative imbalance in silkworms. Under physiological conditions, silkworms maintain redox homeostasis through the coordinated action of antioxidant enzymes including SOD, CAT, and GSH. However, antimony tin oxide exposure, particularly at 3.2 μg/μL, disrupted this balance, leading to a dramatic increase in ROS levels, indicating exacerbated oxidative damage. SOD activity was significantly suppressed, impairing the elimination of superoxide radicals, while GSH content was substantially depleted—consistent with findings from in vitro toxicity studies of ZnO NPs exposure in *Chlorella vulgaris* [44]—and demonstrating parallels with toxicological patterns reported for other metal oxide nanoparticles such as CuO and ZnO, which impair silkworm fitness and antioxidant homeostasis [45]. Notably, ZnO nanoparticles similarly induce oxidative stress and apoptosis in *Bombyx mori* cells [46]. In contrast, TiO_2_ nanoparticles may produce distinct, dose-dependent effects in silkworms [47], indicating that nanoparticle toxicity is material-specific. This comparative framework positions the current ATO findings within broader metal oxide nanoparticle toxicity paradigms. Although CAT activity was elevated, the overall antioxidant capacity was severely compromised. These findings indicate that antimony tin oxide exposure disrupted redox homeostasis in the silkworm midgut. Although only one concentration was used for oxidative stress evaluation in this study, the selected dose (3.2 μg/μL) produced a clear toxic effect with approximately 50% mortality, supporting its use as a biologically effective exposure level. Future studies including multiple exposure levels will be useful to establish a complete dose–response relationship. Collectively, these alterations demonstrate that antimony tin oxide causes profound damage to the silkworm antioxidant defense system. Although oxidative stress biomarkers were measured only at the representative high dose (3.2 μg/μL) in this study, the preceding multi-concentration phenotypic screening demonstrated clear concentration-dependent toxicity. Future studies should extend ROS and antioxidant enzyme measurements to intermediate concentrations to establish a detailed biochemical dose–response relationship.

The midgut was identified as a major target organ of antimony tin oxide toxicity. Histopathological examination revealed severe structural abnormalities, including brush border disruption, epithelial cell exfoliation, and vacuolization. TEM analysis further demonstrated nuclear condensation, mitochondrial swelling or vacuolization, and dilation of the endoplasmic reticulum. These tissue- and cell-level alterations indicate that the integrity of the midgut epithelium was markedly compromised after exposure. Because the silkworm midgut is the primary site for digestion, nutrient absorption, and xenobiotic defense, structural damage to this tissue is expected to impair digestive physiology and thereby contribute directly to larval growth inhibition. The morphological observations therefore provide important structural evidence linking external exposure to internal physiological dysfunction.

Transcriptomic analysis further showed that antimony tin oxide exposure induced substantial molecular disturbance in the larval midgut. GO enrichment revealed a clear functional divergence between up- and down-regulated genes. Up-regulated genes were mainly enriched in cuticle-associated and defense-related functions, including structural constituent of cuticle and immune response, whereas down-regulated genes were primarily clustered in lipid metabolism-related processes, such as triacylglycerol metabolism and fatty acid oxidation. RT-qPCR validation of representative genes, including *GlcNAcase2*, *AGPAT5*, *H9JQS0_BOMMO*, *Chi*, and *H9J6N7_BOMMO*, showed expression patterns consistent with the RNA-seq data. Together, these findings suggest that exposure to antimony tin oxide may shift midgut transcriptional activity toward structural and defensive functions, while simultaneously disturbing lipid metabolic balance. This transcriptional adjustment may represent a stress-responsive adaptation of the larval midgut to nanoparticle-induced injury.

The combined physiological, histological, and transcriptomic evidence suggests that oxidative imbalance and midgut injury are closely linked in the toxic response to antimony tin oxide. Excessive ROS production may damage cellular membranes, proteins, and organelles, thereby contributing to the mitochondrial and epithelial abnormalities observed by TEM and histopathology. At the same time, transcriptional dysregulation of genes associated with cuticle/chitin-related processes and lipid metabolism may further disturb the structural stability and metabolic activity of the midgut. In this context, the up-regulation of structural and defense-related genes may represent an adaptive attempt to maintain epithelial barrier function, whereas the down-regulation of lipid metabolic genes may reflect disrupted nutrient utilization and membrane turnover. Thus, the toxic effects of antimony tin oxide in silkworms are likely not attributable to a single event, but instead arise from the interaction of oxidative stress, epithelial injury, and metabolic dysregulation.

**Figure 9:**
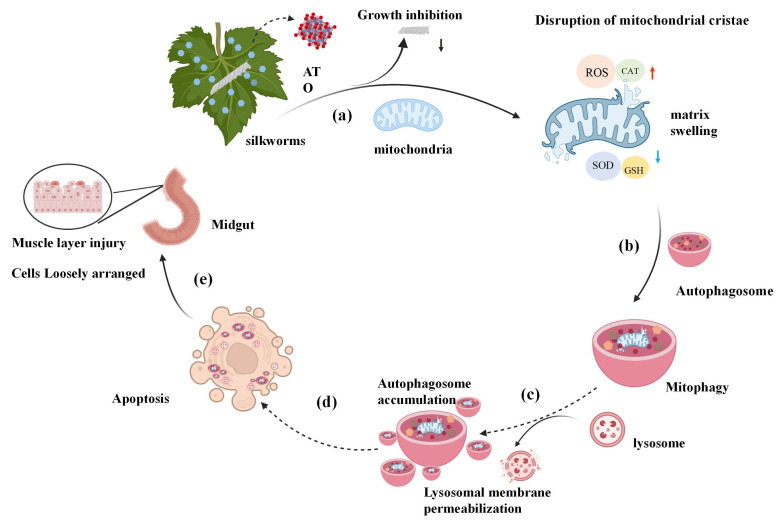
Integrated multiscale mechanisms of antimony tin oxide-induced midgut toxicity in *Bombyx mori*. (**a**,**b**) Antimony tin oxide suppressed larval growth and triggered midgut ROS bursts, concurrent with SOD/GSH inhibition and compensatory CAT up-regulation. Oxidative damage caused mitochondrial cristae fragmentation and matrix swelling, thereby inducing mitophagy. (**c**–**e**) ROS-mediated lysosomal membrane rupture led to enzyme leakage, which may have blocked autophagosome–lysosome fusion and caused autophagosome accumulation. These disruptions, combined with oxidative stress, were associated with cellular destruction phenotypes indicative of apoptosis, ultimately manifesting as brush border destruction and barrier loss. (Black arrows indicate growth inhibition; Red arrows indicate increased activity; Blue arrows indicate decreased activity).

Further studies revealed a molecular cascade initiated with intracellular ROS accumulation (concomitant with SOD/GSH suppression and compensatory CAT elevation), which triggered mitochondrial ultrastructural disintegration. Subsequent activation of mitophagy was counteracted by autophagic flux blockade due to lysosomal membrane leakage, resulting in autophagosome accumulation. This process combined with oxidative stress may be associated with apoptosis-like phenotypes as visually integrated in Figure 9.

In addition to the transcriptomic data, the motif, structural, and interaction analyses of selected genes provide supportive information for understanding the molecular context of the observed response. The conserved domains and predicted interaction patterns of proteins such as AGPAT5, Chi, and H9J6N7_BOMMO suggest that these genes are functionally connected with lipid remodeling, chitin turnover, and digestive or membrane-associated processes. These analyses are useful in highlighting the potential functional relevance of the differentially expressed genes identified in the midgut. However, they should be interpreted as supportive bioinformatic evidence rather than direct mechanistic proof. Further functional validation, such as gene knockdown or overexpression combined with toxicity assays, will be necessary to determine whether these genes play causal roles in antimony tin oxide-induced midgut injury.

Several limitations of the present study should be acknowledged. First, because the exposure model was based on nominal concentrations applied to mulberry leaves, we did not quantify the actual ingested amount of antimony tin oxide, nor did we determine its accumulation in the midgut or its levels in the hemolymph. Therefore, the present conclusions are based on the observed associations between external exposure and downstream physiological, histological, and transcriptomic responses. Second, although oxidative stress biomarkers were measured only at the representative high dose (3.2 μg/μL), the preceding phenotypic screening demonstrated clear concentration-dependent toxicity, and future studies should extend these biochemical analyses across additional exposure levels to establish a more detailed dose–response relationship. In addition, the physicochemical properties of the nanoparticles under the actual exposure conditions, as well as the contribution of dissolved metal species, were not independently assessed in the present work. Future studies combining toxicokinetic analysis, nanoparticle characterization, and soluble metal controls will help to further clarify the mechanisms underlying antimony tin oxide toxicity in silkworms.

Overall, the present study demonstrates that antimony tin oxide causes significant toxic effects in fifth-instar silkworm larvae, manifested as growth inhibition, oxidative stress imbalance, midgut structural damage, and broad transcriptional disturbance. By integrating organismal phenotypes with tissue injury and molecular responses, this work expands current understanding of antimony tin oxide nanotoxicity in insects and highlights its potential ecological risk to sericulture and agricultural environments.

## 5. Conclusions

In conclusion, this study shows that antimony tin oxide exposure causes significant toxic effects in fifth-instar *Bombyx mori* larvae, including reduced survival, impaired antioxidant capacity, and marked midgut injury, as evidenced by histopathological and ultrastructural observations. Transcriptomic analysis identified 239 DEGs, with GO enrichment indicating that the up-regulated genes were mainly associated with cuticle- and chitin-related processes, whereas the down-regulated genes were primarily enriched in lipid metabolism-related pathways. RT-qPCR analysis of representative genes further confirmed the reliability of the transcriptomic results. Together, these findings indicate that antimony tin oxide exposure disrupts midgut structural and metabolic homeostasis in silkworm larvae and highlight its potential risk to sericultural production and the agricultural environment.

## Data Availability

The raw sequence data reported in this paper have been deposited in the Genome Sequence Archive (GSA) at the National Genomics Data Center, China National Center for Bioinformation/Beijing Institute of Genomics, Chinese Academy of Sciences, under accession number CRA039701. The dataset is currently under embargo and will be publicly accessible at https://ngdc.cncb.ac.cn/gsa/search?searchTerm=CRA039701 from 8 March 2026.

## Supplementary Materials

The following supporting information can be downloaded at https://www.mdpi.com/article/10.3390/biology15060508/s1. Figure S1: Multiple sequence alignments of AGPAT5. Conserved amino acid residues are colored white with a black background. Figure S2: Multiple sequence alignments of Chi. Conserved amino acid residues are colored white with a black background. Figure S3: Multiple sequence alignments of H9J6N7_BOMMO. Conserved amino acid residues are colored white with a black background. Table S1: List of differentially expressed genes (DEGs) in response to antimony tin oxide exposure; Table S2: Gene-specific primers used for RT-qPCR analysis; Table S3: Detailed information of *Bombyx mori* genes in different species.
