# Supplementary material for: Physio-Transcriptomic Mechanism of Antimony Tin Oxide Nanoparticle-Induced Midgut Toxicity in Bombyx mori
Source: Biology (Basel). 2026 Mar 22;15(6):508. doi: 10.3390/biology15060508 (PMC13023437; doi:10.3390/biology15060508)
Supplement: Supplementary file 1 [file biology-15-00508-s001.zip › Supplementary File3.11.pdf]

Supplementary Data

Research Article

Physio-Transcriptomic Mechanism of Antimony tin oxide Nanoparticle-Induced Midgut Toxicity in *Bombyx mori*

By Yang Fang[*1,‡,†], Xuan Li[*1,‡], Fengchao Zhang[*2,], Yang Liu[*2], Liang Ma[*1], Liping Chen[*1], Qijun Xie[*1,†]

*1 Hunan Key Laboratory of Biomedical Nanomaterials and Devices, School of Biological Science and Medical Engineering, Hunan University of Technology, Zhuzhou 412007, China

*2 College of Marine Life Sciences, Ocean University of China, Qingdao 266003, China

‡ These authors contributed equally to this work.

† Corresponding authors: Qijun Xie and Yang Fang, Hunan Key Laboratory of Biomedical Nanomaterials and Devices, School of Biological Science and Medical Engineering, Hunan University of Technology, Zhuzhou 412007, China, (Email: qjxie@hnu.edu.cn ; fangyang@hut.edu.cn)

* Corresponding authors: Qijun Xie and Yang Fang

Email: qjxie@hnu.edu.cn; fangyang@hut.edu.cn

Supplementary Table S2: Gene-specific primers used for RT-qPCR analysis

Primers	Primer sequences (5′- 3′)
BMSK0013611-F	*TATTCAGTTGCGTGTCGGAG*
BMSK0013611-R	*GCTTAATCGTGAGTCTTTGG*
BMSK0012668-F	*TATTGCTTAGATAGGTGGAC*
BMSK0012668-R	*TTATTATTATTACTGGTGGT*
BMSK0008588-F	*GTTCTCGTCGCCTAGTTGGT*
BMSK0008588-R	*GTTTCTCGCCGGATTTCTTA*
BMSK0009520-F	*GATGTTTGCTGGATGTTGAT*
BMSK0009520-R	*ATTGCTCTTGAGTTGTTAGG*
BMSK0004232-F	*TAATCAAACTGACAACAAAG*
BMSK0004232-R	*GAAAGAGACAGGAACATACA*
BMSK0003874-F	*TCACTTTCGTCCTTTGGCCT*
BMSK0003874-R	*ACCGAGAATGTATCATCCGT*
BMSK0006293-F	*TAGCCTTTGTCTCGTCGATT*
BMSK0006293-R	*TACTGTTTGTCCGTTTTCCC*
BMSK0007168-F	*ATTTTGTCACACATCCTTCA*
BMSK0007168-R	*CTCTCTCCAATCTACCGCTA*
BMSK0000686-F	*TTGAGCGACAGGAGGTTTTA*
BMSK0000686-R	*TTTTTTTGTGGTCAGGAGGA*
BMSK0006280-F	*ATAAAATGAAGGGAACAAAT*
BMSK0006280-R	*TAAAAGCAGCATACAAACAA*
Rp49-F	*CAGGCGGTTCAAGGGTCAATAC*
Rp49-R	*TACGGAATCCATTTGGGAGCAT*

Supplementary Table S3. Detailed information of Bombyx mori genes in different species

Gene	Species name	Accession number	Sequence Similarity
BMSK0008588 (AGPAT5)	*Maniola hyperantus*	XP_034824087.1	72.50%
	Zerene cesonia	XP_038209095.1	67.32%
	Epargyreus clarus	XP_072936226.1	73.00%
	Hyposmocoma kahamanoa	XP_026323669.1	68.69%
	Vanessa atalanta	XP_047539514.1	58.71%
	Nymphalis io	XP_050359267.1	59.50%
	Melitaea cinxia	XP_045452641.1	65.52%
	Ostrinia nubilalis	XP_063837193.1	63.50%
	Bicyclus anynana	XP_023939060.1	70.44%
BMSK0003874 (Chi)	*Manduca sexta*	XP_030030175.1	84.36%
	Leptidea sinapis	XP_050672426.1	78.04%
	Galleria mellonella	XP_052754063.1	78.71%
	Vanessa tameamea	XP_026497290.2	80.07%
	Anticarsia gemmatalis	XP_075984553.1	80.28%
	Aricia agestis	XP_041982984.1	75.92%
	Epargyreus clarus	XP_072933371.1	81.25%
	Maniola hyperantus	XP_034836040.1	78.90%
	Battus philenor	XP_068619839.1	81.93%
BMSK0007168 (H9J6N7_BOMMO)	*Manduca sexta*	XP_030027522.1	62.27%
	Spodoptera litura	XP_022814449.1	59.69%
	Pectinophora gossypiella	XP_049868595.1	58.95%
	Helicoverpa armigera	XP_049696367.2	57.77%
	Achroia grisella	XP_059046451.1	56.92%
	Ostrinia furnacalis	XP_028173132.1	54.77%
	Vanessa atalanta	XP_047530437.1	57.28%
	Vanessa cardui	XP_046962056.1	56.96%
	Aricia agestis	XP_041982175.1	55.52%

Supplementary Figure S1

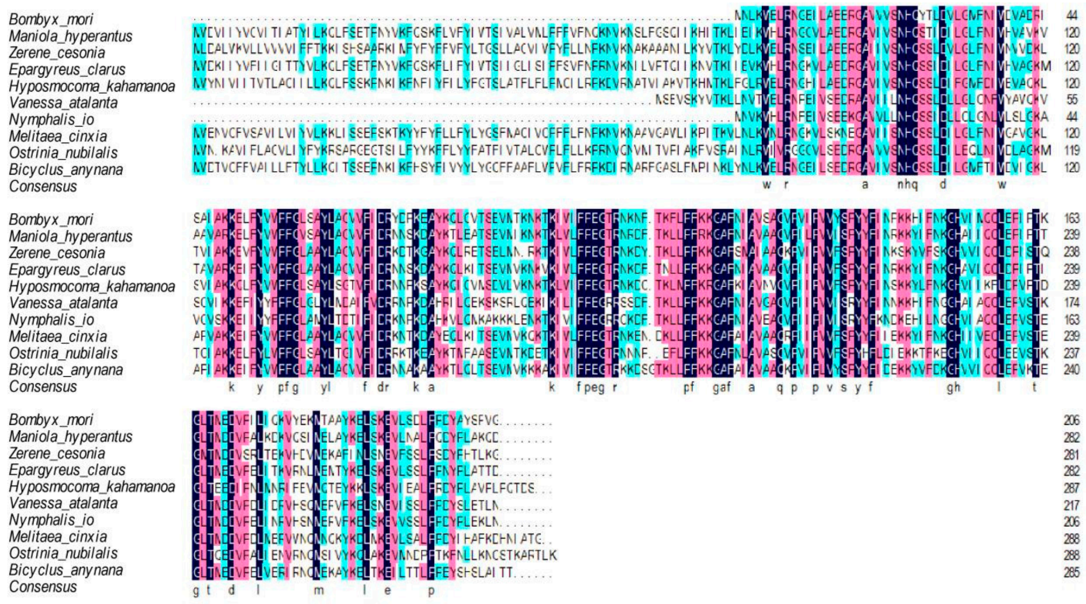

Fig. S1. Multiple sequence alignments of AGPAT5. Conserved amino acid residues are colored white with a black background.

Supplementary Figure S2

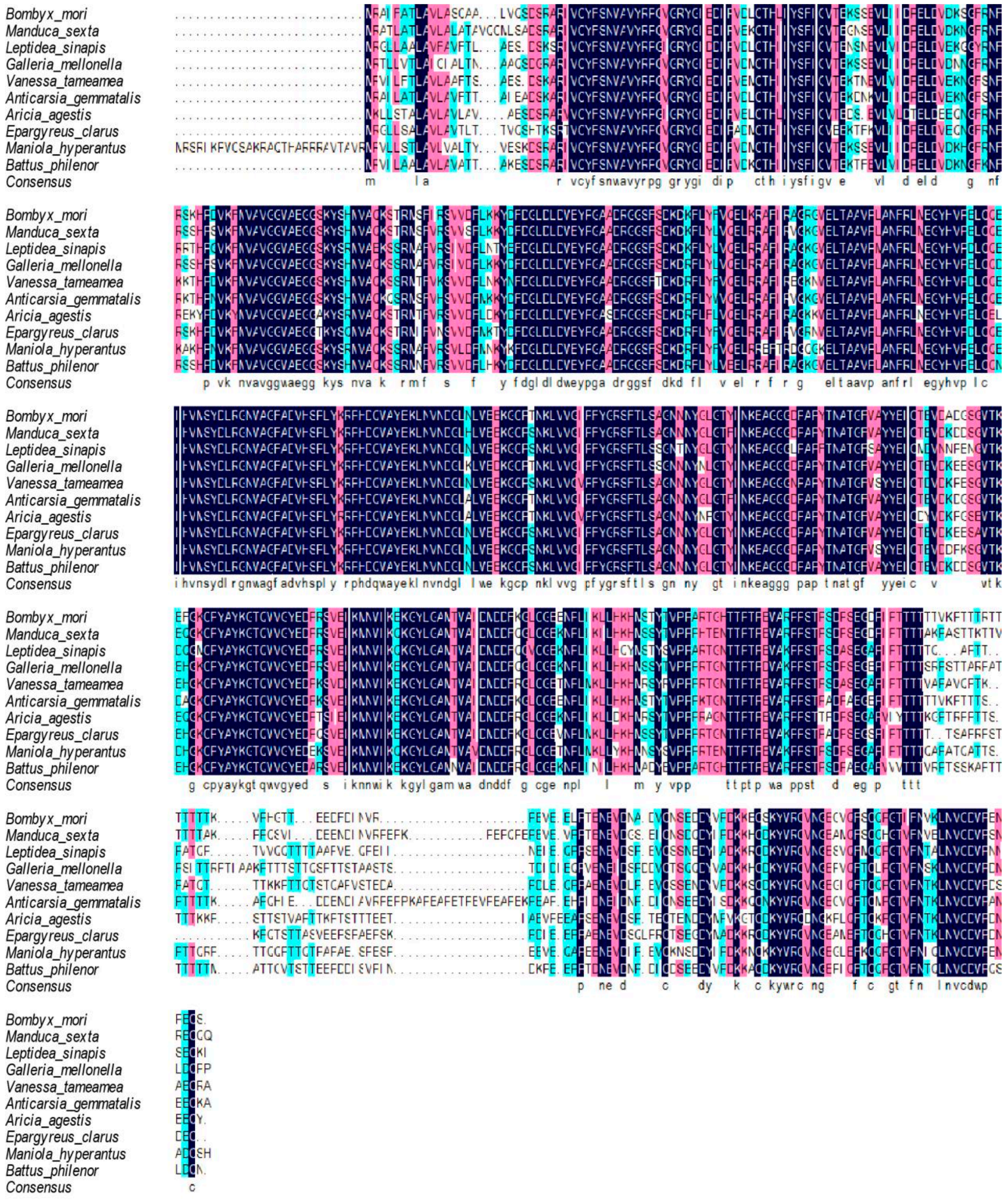

Fig. S2. Multiple sequence alignments of Chi. Conserved amino acid residues are colored white with a black background.

Supplementary Figure S3

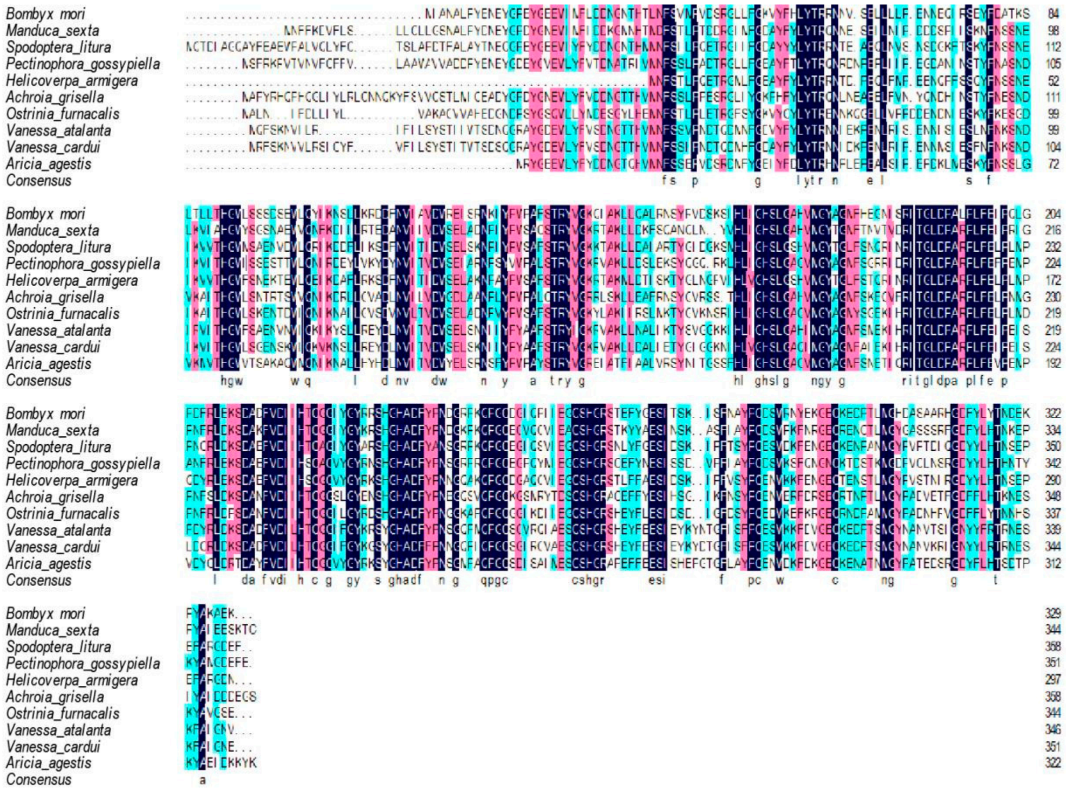

Fig. S3. Multiple sequence alignments of H9J6N7_BOMMO. Conserved amino acid residues are colored white with a black background.
